# Assessing the Impacts of Autonomous Vehicles on Road Congestion Using Microsimulation

**DOI:** 10.3390/s22124407

**Published:** 2022-06-10

**Authors:** Areej Malibari, Akito Higatani, Wafaa Saleh

**Affiliations:** 1 Department of Industrial and Systems Engineering, College of Engineering, Princess Nourah bint Abdulrahman University, P.O. Box 84428, Riyadh 11671, Saudi Arabia; aamalibari@pnu.edu.sa; 2 Hanshin Expressway Co., Ltd., Osaka 553-0003, Japan; higatani-akito@hanshin-exp.co.jp; 3 College of Engineering, Princess Nourah bint Abdulrahman University, Riyadh 84428, Saudi Arabia

**Keywords:** road traffic congestion, car-following models, Helly model, Hanshin area

## Abstract

The introduction of autonomous vehicles has been considered as a possible option for reducing traffic congestion in many transport studies. Many types of models, in particular car-following microsimulation models have been adopted in most studies. The impacts of autonomous vehicles (AVs) on congestion, however, have not yet been concluded. This could be because different researchers use different forms of car-following models to assess these impacts, or because the utilised modelling approaches and their parameters are different in different studies. In particular, two of the important parameters that are associated with car-following models are the used values for maximum acceleration and the average desired time gaps. While the values of these parameters can be adjusted and controlled by the ACC controllers in the AV, they can also be controlled by the users. Therefore, assigning unrealistic values to these parameters could well result in unrealistic conclusions. This paper investigated the impacts of the maximum acceleration and the average desired time gaps on congestion levels using the loss-time indicator. The analysis was carried out on the Hanshin expressway in Japan and was tested and assessed using the Helly (FACC) car-following microsimulation model. This includes estimating the values of the desired time gap from real traffic time-gap distributions. The Hanshin expressway is an urban toll highway of 273 km that extends from Osaka to Kobe, representing the Hanshin area in Japan. The Hanshin highway serves a huge traffic volume that consists of private and freight vehicles that operate within the Hanshin area. This area represents one of three major municipal areas in Japan including Tokyo and Nagoya. A total of 740,000 vehicles per day travel on the expressway. As a result, there is significant congestion on the Hanshin expressway. There have been various plans put in place to ease congestion ranging from building new roads to the implementation of traffic-demand-management measures. However, the predictions of the impacts of such measures do not provide any evidence that they would ease traffic congestion. Other possible measures that could be investigated for easing traffic congestion include technology-based solutions such as autonomous vehicles. The modelling results recommend that the results obtained from microsimulation models should be taken with care, and good attention should be paid to the parameters used and their values in the model. The values assigned to driving-behaviour parameters, the maximum values of acceleration, and the time-gap settings, for example, control the final outcomes of the models.

## 1. Introduction and Previous Work

Several microsimulation models have been calibrated and investigated since the 1950s in order to present and imitate driving behaviour, in which car-following models have been the main component. Examples of car-following models include the Helly model [1], Gipps model (GM) [2], Optimal-Velocity model (OVM) [3], Collision-Avoidance model (CA model) [4], Intelligent-Driver model (IDM) [5], and IDM+ [6]. Microsimulation modelling and car-following models have been widely used to assess the impact of autonomous-vehicle operation, simulation and driving [1,2,3,4,5,6,7,8,9,10,11,12,13]. The impact of autonomous vehicles on congestion has not yet been concluded. Firstly, many researchers used different forms of car-following models to assess their impacts on congestion. Secondly, the modelling approaches and parameters varied significantly in different studies.

While some researchers claim that there will be no congestion in the future with the increasing use of autonomous vehicles [14,15], others claim otherwise [16,17]. There are two main reasons for this dilemma. Firstly, there is a lack of data and knowledge on the behaviour of autonomous vehicles in congested networks; in particular, the acceleration and deceleration behaviour of autonomous vehicles at the low-speed range have not been studied since the full-range ACC (FACC) was just commercialised a few years ago. Secondly, there are issues with the parameters used in these models and their values.

Therefore, when studying the behaviour of autonomous vehicles, it is important to pay attention to the parameters used in the analysis and their consequences. Car-following models are extensively used to model and assess the behaviour of autonomous vehicles in the literature. Central to these models are the maximum values of acceleration and the desired time gap. The values assigned to these parameters immensely affect the results of the models. For example, previous research applied a maximum-acceleration value of 0.6 m/s^2^ [15], which in many cases resulted in a significant reduction in congestion. This maximum-acceleration value, however, is less than half of the general maximum acceleration of non-autonomous vehicles, which is usually in the range of 1.6 m/s^2^. On the other hand, other studies used higher values of maximum acceleration that are closer to the values associated with non-autonomous vehicles. For example, the IDM+ model used a value of 1.4m/s^2^, which resulted in higher traffic congestion with autonomous vehicles. Similarly, the desired time gap is a very important parameter to control vehicle behaviour. The values of these parameters can be adjusted and controlled by the ACC using sensors or manually by the drivers. This is particularly true of the less advanced autonomous vehicles. Therefore, setting unrealistic values for such parameters might result in unrealistic benefits in terms of reducing congestion and improving network conditions.

Microsimulation-modelling tools that are used to predict and replicate the driving behaviour of vehicles are mainly based on car-following models. Three main requirements are essential in any model to be able to represent autonomous-vehicle behaviour: the desired time gap, the collision-avoidance system, and the sensor detection range (Table 1). Some models are limited in terms of representing the varying desired gap that is related to vehicle speed [18]. The GM model, for example, does not include the desired time gap or desired gap. Therefore, it has limitations in terms of representing AV behaviour.

## 2. Car-Following Models

### 2.1. Introduction

There are a number of car-following models that have been developed and applied to many case studies. The Helly model [1], Gipps model (GM) [2], Optimal-Velocity model (OVM) [3], Collision-Avoidance model (CA model) [4], Intelligent-Driver model (IDM) [5], and IDM+ [6] are the most widely used models to assess car-following behaviour in the literature. In particular, the Helly model is very popular and very commonly used since its structure and processing are manageable and easily adaptable. Car-following models include many parameters. It is apparent that each model was developed for a certain case study; hence, the set of parameters are calibrated using a specific set of experimental data and therefore, their applicability to other case studies has to be justified and taken cautiously. While many studies have compared and evaluated a number of car-following models in order to assess their capabilities, there are no defined set of recommendations to date on which model is best in which circumstances; see for example [16,17,18]. Examples of car-following models include Treiber et al. [5,14], who applied the IDM model to replicate the impacts of driving autonomous vehicles on traffic flows, and Suzuki et al. [15], who modelled the driving behaviour of autonomous vehicles by applying the IDM+ to represent human drivers with different parameters. Other types of car-following models include, for example, the Intelligent-Driver model (IDM), IDM+, and Alexandros E. Papacharalampous’s model (A.E.P.), which have been assessed in this study. Arne Kesting et al. [16] suggested that the IDM-type controller, whose parameters are different from those of nonautonomous vehicles, is best to use.

Three main requirements are essential in any car-following model to be able to represent autonomous-vehicle behaviour: the desired time gap, the collision-avoidance system, and the sensor detection range (Table 1). Some car-following models are limited in terms of representing the varying desired gap that is related to vehicle speed [13]. The GM model, for example, does not include the desired time gap or desired gap. Therefore, it has limitations in terms of representing AV behaviour.

In this paper, the methodology is presented in Section 2. This includes a brief description of the car-following models that were used in this research and their assessment. Section 3 presents the case study that was investigated in this research. The analysis of the modelling results is presented in Section 4. Section 5 concludes the findings and suggests further research.

### 2.2. Car-Following Models in This Study

In this study, three models were used to carry out this assessment:

1. The Helly model, as a car-following model, to model autonomous-vehicle behaviour;
2. An IDM+ model to model non-autonomous-vehicle behaviour;
3. A VISSIM model, which is a traffic-microsimulation tool with an external car-following model. This is because it was not possible to access or use the VISSIM’s car-following model and it was not possible to utilise it to model autonomous vehicles. The VISSIM model was also used to model the lane-changing behaviour of the entire corridor. A brief description of each of these models is discussed in this section.

#### 2.2.1. Helly Model

The Helly model is a widely used and applied car-following model in which the vehicle acceleration is controlled by the distance gap and speed difference, as in Equation (1):

(1) $$\frac{{\mathrm{dv}}_{i}\left(t\right)}{\mathrm{dt}}=\alpha ∆v_{i}\left(t\right)+\beta \left(∆x_{i}\left(t\right)-\left(s_{0}+v_{i}\left(t\right)T\right)\right)$$

where:

I is an index for the vehicle;

v_i_(t) is the speed;

Δv_i_(t) is the relative speed (with respect to the preceding vehicle);

Δx_i_(t) is the distance gap;

T is the desired time gap;

s_0_ is the minimum gap at standstill;

α is the sensitivity parameter with respect to relative speed Δv_i_(t);

β is the sensitivity parameter with respect to the difference between the current gap and the desired gap.

It is important to note that the Helly-model-type controllers have no collision-avoidance concept. Therefore, braking behaviour such as emergency stopping cannot be adequately represented in this model. The Helly model was assessed and a newer version of this Helly-model-type controller was formulated as follows (Equation (2)) [8,17]:

(2) $$\frac{{\mathrm{dv}}_{i}\left(t\right)}{\mathrm{dt}}=\min\left[k\left(v_{0}-v_{i}\left(t\right)\right),(\alpha ∆v_{i}\left(t\right)+\beta \left(∆x_{i}\left(t\right)-\left(s_{0}+v_{i}\left(t\right)T\right)\right)\right]$$

where v_0_ is the desired speed and *k* is the sensitivity parameter with respect to the difference between the desired speed and current speed. The rest of the parameters are the same as explained. The first part of the formula was added in order to distinguish between free and following driving behaviour. It is important to note that the Helly-model-type controllers have no collision-avoidance concept. Therefore, braking behaviour such as emergency stopping cannot be adequately represented in this model.

The updated Helly model is referred to as the Helly (FACC) model. The Helly model was updated to be able to deal with the collision-avoidance system [18]. The constraints of speed and acceleration, the concept of sensor detection range, and enhancing brake function were added to the model as in Equations (3)–(5) below.

The Helly (FACC) model is formulated as follows:

(3) $$\frac{{\mathrm{dv}}_{i}\left(t\right)}{\mathrm{dt}}=\{\begin{array}{c} \delta \left(\alpha ∆v_{i}\left(t\right)+\beta \left(∆x_{i}\left(t\right)-\left(s_{0}+v_{i}\left(t\right)T\left(t\right)\right)\right)\right)\;\;\mathrm{if}\;∆x_{i}\left(t\right)\leq s_{i}^{\mathrm{ACC}}\\ \gamma \left(v_{0}-v_{i}\left(t\right)\right)\;\;\;\;\;\;\;\;\;\;\;\;\;\;\;\;\;\;\;\;\;\;\;\;\;\;\;\;\;\;\;\;\;\;\;\;\;\;\;\;\;\;\;\;\;\;\;\;\;\;\;\;\;\;\;\;\mathrm{if}\;∆x_{i}\left(t\right)>s_{i}^{\mathrm{ACC}} \end{array}$$

(4) $$T\left(t\right)=\min\left(k_{1,\mathrm{setting}}+k_{2,\mathrm{setting}}/v_{i}\left(t\right),{\;k}_{3,\mathrm{setting}}\right)$$

(5) $$\delta =\{\begin{array}{c} \max\left(\max\left(\frac{v_{i}^{2}}{2∆x_{i}b_{i}}-\frac{v_{i-1}^{2}}{2∆x_{i}b_{i-1}},\;0\right)+\frac{c}{∆x_{i}},1\right)\;\;\;\;\;\;\;\;\mathrm{if}\;a<0\\ \;\;\;\;\;\;\;\;\;\;\;\;\;\;\;\;\;\;\;\;\;\;\;\;\;\;\;\;\;\;\;\;\;\;\;\;\;\;\;\;\;\;\;\;\;1\;\;\;\;\;\;\;\;\;\;\;\;\;\;\;\;\;\;\;\;\;\;\;\;\;\;\;\;\;\;\;\;\;\;\;\;\;\;\;\;\;\;\;\;\;\mathrm{if}\;a\geq 0 \end{array}$$

with

(6) $$0\leq v_{i}\left(t\right)\leq v_{\max}$$

(7) $$a_{\min}\leq \frac{{\mathrm{dv}}_{i}\left(t\right)}{\mathrm{dt}}\leq a_{\max}\;$$

where:

α is the sensitivity parameter with respect to relative speed Δv_i_(t), and equals 0.5;

β is the sensitivity parameter with respect to the difference between the current gap and the desired gap, and equals 0.125;

γ is the onboard sensor detection range and equals 0.2;

$s_{i}^{\mathrm{ACC}}$ is the minimum gap at standstill and equals 120 m;

a_min_ is minimum acceleration and equals −8 m/s^2^;

a_max_ is maximum acceleration and equals 0.6 m/s^2^;

δ is defined as the safety risk.

The values of the parameters that are related to the time-gap-control strategy, ${\mathrm{Nk}}_{2,\mathrm{setting}}$ and $k_{3,\mathrm{setting}}$, are given in Table 2.

In the modified model, Equations (3) and (4) are the same as the original equation in the Helly model. Equation (3) represents the acceleration of the vehicle and Equation (4) represents the strategy for time-gap control. Equation (5) is the add-on function to represent the collision-avoidance system in order to include a brake function in the model with the parameter *δ* defined as the safety risk. This function is introduced in the model as a function of the gap settings, *v_i_*_,_ is the speed, Δ*x_i_(t)* is the distance gap, *b_i_* and *b_i-1_* are decelerations equal to 0.3 G (2.97 m/s^2^), and *c* is 4 m. The concept of safety risk is grounded on the Mazda algorithm [19,20,21,22] and is represented in Equations (6) and (7). In this research, we applied the Helly (FACC) [18] model to assess the Hanshin corridor and assess the impact of using autonomous vehicles on the congestion level.

#### 2.2.2. The IDM+ Model

The second utilised model in this study is a traffic-flow model known as the Intelligent-Driver model (IDM+). This is a time-continuous car-following model that is suitable for use in simulating urban and motorway traffic [5]. It was developed to improve car-following-behaviour modelling over the existing car-following models and it is a typical model to represent non-autonomous-vehicle behaviour. It has been tested in many network analyses and investigations. The model is represented as follows (Equation (8)):

(8) $$\frac{{\mathrm{dv}}_{i}\left(t\right)}{\mathrm{dt}}=a*\min\left(1-{\left(\frac{v_{i}\left(t\right)}{v_{0}}\right)}^{4},1-{\left(\frac{s^{*}\left(v_{i}\left(t\right),∆v_{i}\left(t\right)\right)}{∆x_{i}\left(t\right)}\right)}^{2}\right){\;\;s}^{*}\left(v_{i}\left(t\right),∆v_{i}\left(t\right)\right)=s_{0}+v_{i}\left(t\right)T+\frac{v_{i}\left(t\right)∆v_{i}\left(t\right)}{2\sqrt{\mathrm{ab}}}$$

where:

a is 1.4;

b is 2.1;

T is 1.55 s.

The rest of the notations are the same as in Equations (1)–(7) above. The values of the parameters, a and b, are the same values as those suggested by Bernat Goñi-Ros et al. [23]. The value of T, desired time gap, was obtained from the real traffic time-gap distribution.

#### 2.2.3. VISSIM Model

In order to model the impacts of vehicles’ movement on traffic, a traffic-modelling software is needed. VISSIM is a microscopic multi-modal traffic-flow model with a simulation software. This model allows the use of other modular modelling software as appropriate. For example, in this study VISSIM software was used with the car-following components from the updated Helly model (FACC) and the IDM+ model for the AV and non-AV, respectively.

## 3. Methodology

### 3.1. General Description of Case Study

**Figure 1:**
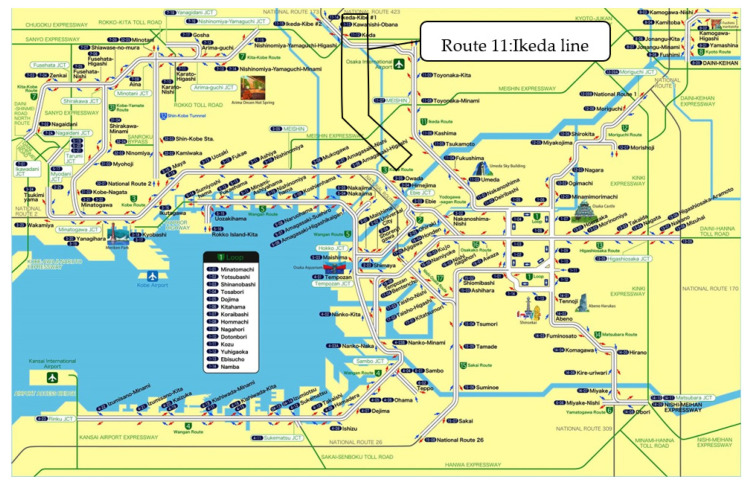
Hanshin expressway network.

The Hanshin area, Tokyo and Nagoya are three of the most major and congested metropolitan areas in Japan. The Hanshin expressway is an urban toll express highway with a length of 273 kms. It extends from Osaka to Kobe (the Hanshin area in Japan; Figure 1) [20].

The Hanshin express highway services private cars and freight traffic movement with a total daily traffic of 740,000 vehicles. As a result, there is significant congestion on the Hanshin expressway. On the Hanshin expressway, ultrasonic vehicle detectors have been installed every 500 m. These detectors and other equipment collect the traffic-flow data and send them to central processing units. The Ikeda line (Route 11), one of the busiest routes in the Hanshin expressway network. runs from north to south, connecting to the loop route in central Osaka. There are plans to construct some new parts of the expressway in the Hanshin expressway network in the future. However, the predictions show that even with these new constructions, traffic congestion will remain a major problem. Therefore, the impact of autonomous vehicles on this congestion is a major concern.

### 3.2. Traffic Characteristics of the Ikeda Line

**Figure 2:**
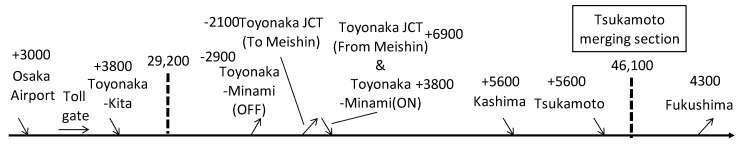
Average traffic volume in 2016.

The Ikeda line is a two-lane road with an operational speed of 60 km/h. Significant traffic volume joins with this line from two interurban expressways (Chugoku expressway and Meishin expressway) in order to reach the centre of Osaka. Figure 2 shows the average weekday traffic volume in 2016. The maximum traffic volume on this line reaches over 46,000 vehicles per day. The morning peak time is from 7 to 9 am and the evening peak time is from 3.00–7.00 pm. The hourly traffic volume at the peak time reaches around 7% of the daily traffic volume. The drivers use the Ikeda line to quickly travel to the centre of Osaka by crossing the Yodo River, which is one of the largest rivers in the Hanshin area between the northern Osaka area and the centre of Osaka. On the Ikeda line, there are other local roads along this corridor; however, they are not capable of dealing with the traffic demand.

**Figure 3:**
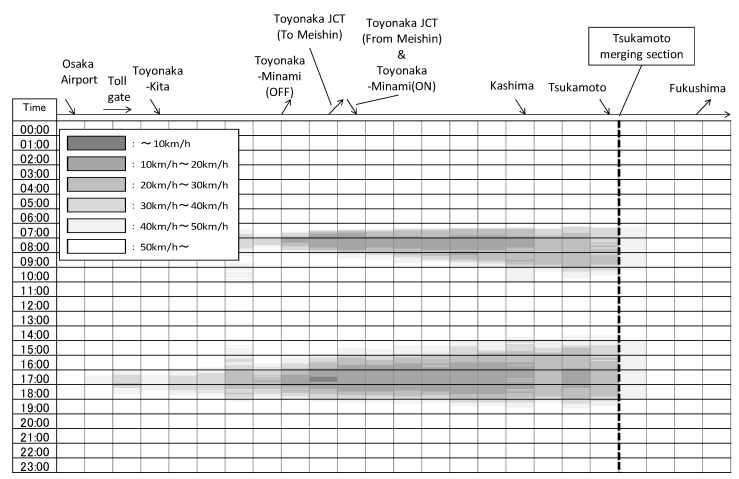
Velocity contour map (November in 2015).

**Figure 4:**
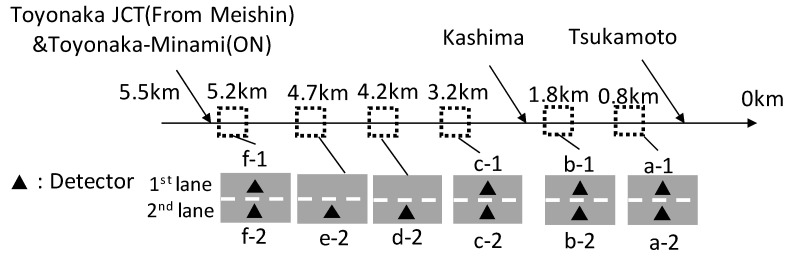
Simulation network.

The length of the study section of the network was 5.5 km and spanned from the Tsukamoto merging section to around the Toyonaka junction (Figure 3). The network had 10 detectors (a-1 to f-2) to compare the simulation results to the real traffic data. Figure 4 shows the histogram of the time gap that was composed of values under 3 s. The mean value of the distribution was 1.55 s. The time-gap distribution came from vehicle-trajectory data, which were created using video-image data around the Tsukamoto merging section. The details of the vehicle-trajectory data can be seen on the ZTD (Zen Traffic Data) website [24].

### 3.3. Analysis of Traffic Characteristics

Severe traffic congestion on the Hanshin corridor is caused by vehicles that merge onto the expressway from the Tsukamoto on-ramp. There are two traffic peaks: from 7.00–9.00 am and 3.00–7.00 pm. Figure 3 shows the weekday average speed transition and the two daily traffic peaks. The congestion begins at the Tsukamoto merging section and extends to the Toyonaka–Minami on-ramp, which includes merging vehicles from the Meishin expressway. There are three on-ramps between the Toyonaka junction and the Tsukamoto merging section, but no off-ramp. That means that once drivers enter this section, no matter how severe the traffic congestion becomes, drivers are trapped and they cannot exit the expressway until they reach the Fukushima off-ramp. In order to control the traffic demand and calm this traffic congestion, a number of measures have been implemented, including:

1. Increasing the toll fee;
2. Implement toll discount during early morning or late evening time periods;
3. Operate traffic control ramps;
4. Construct new roads and bridge;
5. Increasing the toll fee is showing good impact on reducing traffic congestion.

Implementing a discounted toll fee outside the peak period seems to be an effective measure. However, in practice this measure did not have many impacts on reducing traffic congestion, since most of the Hanshin expressway peak traffic is commuting, working, or freight traffic, and it is not very practical to expect significant impacts on this type of traffic. Traffic-control ramps have also been implemented, but did not have major impacts on traffic congestion on the corridor. Obviously, constructing new roads and bridges might have greater impacts; however, this effect is the most difficult to measure.

Microsimulation modelling was utilised to assess and investigate the impact of autonomous vehicles on the traffic congestion of the network of the Hanshin corridor. As discussed, three models were used to carry out this assessment: A VISSIM model, which is a microsimulation tool with a an external car-following model, as well as the Helly (FACC) and IDM+ models.

## 4. Modelling Process

The length of the modelled network was 5.5 km and began at the Tsukamoto merging section and ended around the Toyonaka junction. The network had 10 detectors (a-1 to f-2) to compare the simulation results to the real traffic data. Therefore, it was possible to use the add-on modules in this study (Figure 4). One of the advantages of using the VISSIM model is being able to use other driver models.

The aim of the modelling process was to assess the impacts of utilising different values of the parameters (maximum acceleration and time-gap setting) on the modelling results. Two different values for the maximum acceleration (a_max_ = 0.6 m/s^2^ and 1.4 m/s^2^), representing typical observed values for current non-AV traffic and anticipated values for AV traffic, respectively, were used. The composition of real autonomous-vehicle time-gap settings were calculated from real traffic-flow data (33% very short, 29% short, 24% middle, and 14% long).

A total of ten case scenarios were generated and investigated based on different compositions of traffic and percentages of autonomous vehicles, as well as the different values of maximum acceleration and time-gap settings. The composition of traffic and % AV ranged from 0% AV and 100% non-AV to 100% non-AV and 0% AV. Table 3 summarises the case scenarios.

**Figure 5:**
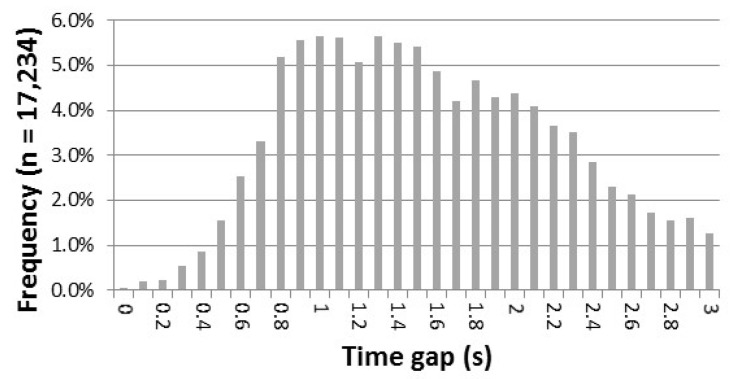
Distribution of the time gap.

From the table, it is apparent that case scenario 1 represents a case of 100% non-autonomous vehicles while case scenario 10 represents a case of 100% autonomous vehicles. Case No. 2 represents a scenario where 95% of vehicles are non-AVs and only 5% are AVs. Case No. 3 represents a scenario where 90% of vehicles are non-AVs and only 10% are AVs, and so on up until case No. 6, which represents a scenario where 50% of vehicles are non-AVs and 50% are AVs. In cases 2–7, the maximum-acceleration value a_max_ = 0.6m/s^2^. The composition of real autonomous-vehicle time-gap settings in cases No. 2 to No. 7 was calculated from real traffic-flow data (33% very short, 29% short, 24% middle, and 14% long). The average values of the distribution were used as the desired time gap of non-autonomous vehicles, and it was assumed that drivers’ preferences of time gaps were maintained as they were with the non-AVs. Then, the values of the proportion were calculated from the distribution in Figure 5.

Case No. 8 represents a scenario where 75% of vehicles are non-AVs and 25% are AVs, and case No. 9 represents a scenario where 100% of vehicles are non-AVs, with no AVs. In both scenarios (8 and 9), the maximum-acceleration value used was a_max_ = 1.4 m/s^2^ and the composition of real autonomous-vehicle time-gap settings was the same as in cases No. 2 to No. 7. In order to create the composition of the autonomous-vehicle time-gap setting in cases No. 2 to No. 7, it was assumed that drivers’ preferences of time gaps were maintained as present. Then, the values of the proportion were calculated from the distribution in Figure 5.

The IDM+ model is represented as in equation (8) included below for completion):

$$\frac{{\mathrm{dv}}_{i}\left(t\right)}{\mathrm{dt}}=a*\min\left(1-{\left(\frac{v_{i}\left(t\right)}{v_{0}}\right)}^{4},1-{\left(\frac{s^{*}\left(v_{i}\left(t\right),∆v_{i}\left(t\right)\right)}{∆x_{i}\left(t\right)}\right)}^{2}\right)\;{\;s}^{*}\left(v_{i}\left(t\right),∆v_{i}\left(t\right)\right)=s_{0}+v_{i}\left(t\right)T+\frac{v_{i}\left(t\right)∆v_{i}\left(t\right)}{2\sqrt{\mathrm{ab}}}$$

In this microsimulation, traffic flow during two hours from 2 pm to 4 pm on 14th December in 2016 was reproduced. Traffic congestion started from Tsukamoto merging section at around 3 pm. Then the congestion spread over towards Toyonaka junction.

## 5. Analysis of the Results

### 5.1. Analysis of Loss Time

**Figure 6:**
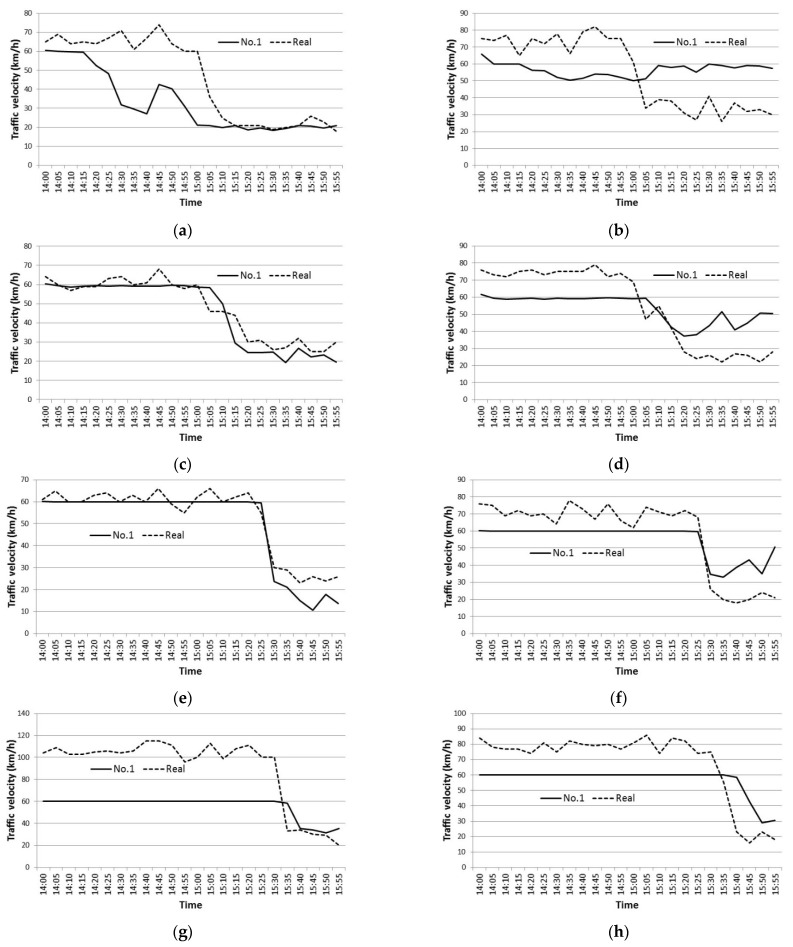

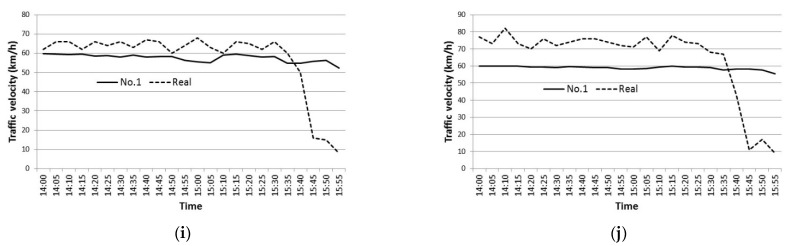
(**a**) Velocity in 1st lane at a-1 (No. 1); (**b**) Velocity in 2nd lane at a-2 (No. 1); (**c**) Velocity in 1st lane at b-1 (No. 1); (**d**) Velocity in 2nd lane at b-2 (No. 1); (**e**) Velocity in 1st lane at c-1 (No. 1); (**f**) Velocity in 2nd lane at c-2 (No. 1); (**g**) Velocity in 2st lane at d-2 (No. 1); (**h**) Velocity in 2nd lane at e-2 (No. 1); (**i**) Velocity in 1st lane at f-1 (No. 1); (**j**) Velocity in 2nd lane at f-2 (No. 1).

As discussed, the network had ten detectors (a-1 to f-2) that were used to compare the simulation results to the real traffic data. The result of case No. 1 compared to the real traffic data is discussed in this section. Figure 6a–j show the simulation results of traffic velocity compared with the real traffic data. The results were obtained by averaging three out of five possible results, excluding both the best and worst results. In this simulation, the maximum velocity was set at 60 km/h, which exhibited differences from the observed real maximum traffic velocity, which in some cases reached 80 km/h and 100 km/h in the 2nd lane. It should be noted that the maximum velocities in the 1st lane were observed to be lower than in the 2nd lane. Traffic congestion patterns in both lanes were similar, however, with congestion beginning around 3 pm, then reaching the Kashima on-ramp after 30 min, before reaching the Toyonaka junction around 4 pm.

From Figure 6a, traffic velocity begins to decrease at the a-1 detector location at around 3.00 pm, similar to real traffic-data behaviour, although there seems to be a temporary reduction in velocity at about 2:30pm. Then, at around 3 pm, the traffic congestion begins to spread over the downstream link. The simulated timing of the speed reduction concurs with that of the real traffic data. Therefore, this simulation seems to be reproducing the real traffic flow accurately. As seen from Figure 6g–i, traffic congestion reaches a distance of 4.7 km, but does not quite reach the Toyonaka junction. Hence, the simulated congestion level seems to be a little lower compared to the real traffic data.

Figure 6b,d,f show that the velocity decreased to just under 30 km/h in reality, while the velocity in the simulation decreased to at most 40 km/h. That is because lane-changing behaviour in the simulation differed from that of reality. Lane changing is restricted around all the three on-ramps in order to organise traffic flow in both the simulation and in reality. However, in reality some vehicles change lanes. Some drivers even change lanes immediately before or after the restricted areas in order to avoid merging vehicles or to avoid congestion, and therefore most of the vehicles perform non-smooth lane changes that may cause shockwaves toward the downstream links. These phenomena seem to be the main cause of the differences between real and simulated traffic. However, these were difficult to reproduce in this microsimulation.

**Figure 7:**
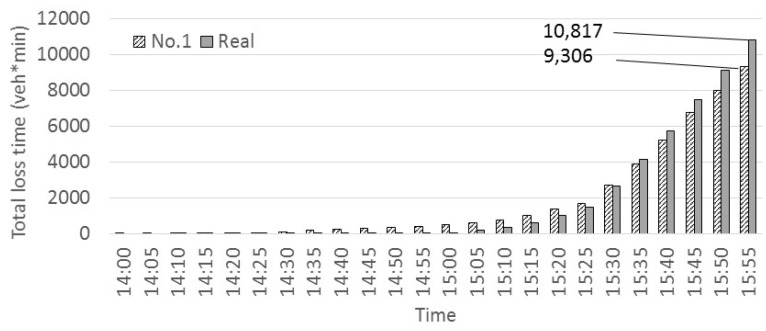
Comparing loss time (No.1 and real data).

Figure 7 shows the comparison of the total loss time for a two-hour period between the observed data and the simulated data for case No. 1.

The indicator of the congestion that was adopted in this study is the loss time. However, other indicators are also possible for future research. The loss time was calculated every 5 min using Equation (9) below:

(9) $$Loss\;time=Q*\left(\frac{L}{V}-\frac{L}{V_{r}}\right)*60$$

where *Q* is the traffic volume, *V* is the traffic velocity, *V_r_* is the regulation speed and *L* is the length of the section. The trend between simulated case No. 1 and the real data is similar. It should be noted, however, that although the total value of the loss time simulated in case No. 1 was 14% less than in the observed data, it was as a result of the congestion not reaching the Toyonaka junction, as discussed earlier. However, the overall trends produced by the simulation very nicely matched the real data. It is possible, therefore, to claim that the simulation in this investigation seems to have sufficient reproducibility.

### 5.2. Analysis of Impacts on Congestion

**Figure 8:**
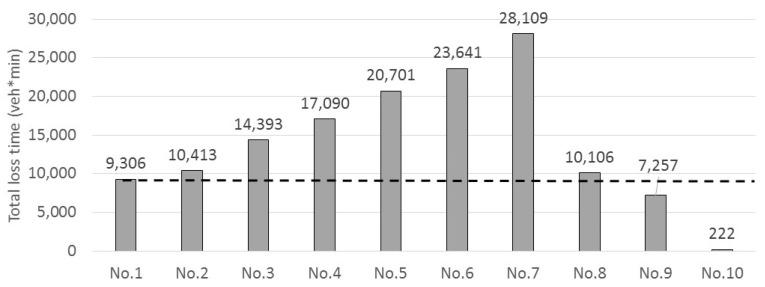
Comparing loss time (No. 1 to No. 10).

Figure 8 presents the total loss time with different case scenarios (from case No. 1 with no AVs to No. 10 with all AVs). From the figure, it appears that, excluding the first and last scenarios, the total loss time increased as the percentage of AV in traffic increased. In scenario 5, where 25% of traffic is Avs, traffic congestion appears to double.

The reason for these results is that the assigned average desired time gap of autonomous vehicles was around 1.7 s, which was longer than the value of the non-autonomous-vehicle desired time gap of 1.55 s. Moreover, the autonomous-vehicle speed recovery after the speed reduction was slower than a non-autonomous vehicle because the maximum acceleration of the autonomous vehicle was set as 0.6 m/s^2^. These two parameters doubly affected the level of the resulting congestion in the network. Changing the values of the parameters will result in changing results. For example, the maximum-acceleration value was changed to 1.4 m/s^2^ from 0.6 m/s^2^ in case No. 8. With 25% of the fleet being AVs, which is similar to case No. 5, the loss time was much lower than in case No. 5. In fact, it was the same value as that calculated for case No. 1. Interestingly, the maximum-acceleration value of 1.4 m/s^2^ is the same parameter used in the IDM+.

Compared with this analysis, the acceleration ability of the Helly (FACC) model is superior to that of the IDM+ according to the results reported in [18]. Hence, the autonomous-vehicle speed recovery was faster than non-autonomous vehicles, as in case No. 8. This is the reason for the observed reduction in loss time in case No. 8.

Other observations from this analysis include, for example, case No. 8, since 75% of all the vehicles are non-autonomous vehicles, if follows that if the leading vehicle is a non-autonomous vehicle, the following autonomous vehicle will no doubt follow the non-autonomous vehicles. Therefore, even with a possible higher maximum acceleration, the desired time gap will be the controlling factor in this case.

Comparing the results of case nos. 8 (with 25% AVs) and 9 (with 100% AVs) shows that the loss time in case No. 9 was lower than in case No. 8, and much lower than in case No. 1. This is because under the conditions that most of the traffic is AVs, increasing the maximum acceleration will significantly contribute to congestion and can overcome the influence of the desired time gap.

Finally, traffic congestion did not occur in case No. 10, in which all traffic is AVs. The reason is that the desired time-gap setting of all the vehicles was set to be very short. That means they follow their leading vehicles with the desired time gap from 1 s to 1.2 s. Autonomous vehicles accelerated slower than non-autonomous vehicles because of the maximum acceleration, 0.6 m/s^2^, in case No. 10. Nevertheless, reducing the average time gap seems to have a greater impact on congestion than increasing the maximum acceleration.

## 6. Discussions and Conclusions

In this study, the impact of autonomous vehicles (AVs) on congestion was observed and investigated using the Helly (FACC) model and loss time as an indicator. Central to the analysis were the assigned values of the maximum acceleration and the desired time gaps. From the results, it seems that the only case where a reduction in congestion was obtained is when all the traffic is autonomous (100% AVs). When the non-AV percentage in traffic increases, congestion will occur, and could well be worse than that of all-non-AV traffic.

Previous research applied a maximum-acceleration value of 0.6 m/s^2^ [15] to be associated with AV traffic, which resulted in a reduction in traffic congestion. This maximum-acceleration value, however, is lower than the common maximum acceleration of non-autonomous vehicles (1.6 m/s^2^). This could be a non-realistic value to be assigned to autonomous vehicles for two reasons. Firstly, the drivers of the AV could have the control to adjust the maximum acceleration of the vehicles and use similar values to what they are currently using in non-AVs (in the range of 1.6 m/s^2^). The other related factor is the overall composition of traffic in terms of the percentage of AVs. It should be noted that the presence of non-AVs in the traffic will affect the overall performance of the traffic and congestion. This will depend on what vehicle is leading the traffic convoy, etc.

The second parameter that was investigated in this study is the longer average desired time gap of autonomous vehicles and their impacts on congestion. There are not many previous studies that have investigated the impacts of varying time-gap settings on traffic congestion and loss time, especially when the drivers have control over selecting and adjusting these time gaps. This study assessed the impacts of using a real time-gap distribution from real traffic-flow data on the loss time and congestion. The average value of the distribution was used as the desired time gap of non-autonomous vehicles. Four settings of the desired time gap were prepared for autonomous vehicles (very short, short, middle, and long). The proportion of settings was calculated on the basis that drivers of autonomous vehicles would use similar time-gap setting as they used to drive non-autonomous vehicles. As a result, the average desired time gap of autonomous vehicles increased by around 10% and became the cause of increasing traffic congestion.

The findings from this research suggest that these two parameters will affect the behaviour of autonomous vehicles and the overall level of congestion in the network. Further research into the behaviour of AVs is needed in terms of the selection of time-gap values when they begin driving on the roads. The drivers might set the time gaps lower than they currently do with non-AVs in order to save time and reduce delays. On the other hand, if driving autonomous vehicles becomes an opportunity to reduce driving times and to allow drivers to engage in other activities, then time-gap values might be set higher to guarantee safety and comfort.

Time-gap settings and manuals for different vehicles are available, and these should be taken into account when investigating and assessing time-gap values and their impacts on driving behaviour in order to guarantee more realistic results.

Finally, in order to reduce traffic congestion, the utilisation of connecting technologies is, of course, the way forward. Connected Auto Cruising Control (CACC) is an essential component in traffic-management programs. This option has not been considered in this paper; however, it is a suggestion for further future research. Controlling speed reduction can only be successfully achieved if all other vehicles are connected, so each vehicle’s speed information and lane-changing information is provided to all other vehicles at any time interval. Additionally, dampening of the shockwave might be possible using the CACC technology. Nevertheless, there will always be problems related to the penetration rate of connected vehicles. Under the circumstance of low penetration, connected vehicles are forced to rely on the information provided externally, therefore they cannot accurately perform all functions. Thus, improving the penetration rate is a critical requirement for the successful utilisation of the above-discussed concepts. It is unavoidable, however, that any alarming news about autonomous-vehicle incidents in the media would hinder auto-driving-technology progression, even if only briefly. CACC is needed to simultaneously improve both technology and reliability in order for the autonomous vehicles to become a successful option for improving congestion and providing a desirable driving alternative for drivers.

## Figures and Tables

**Table 1:** The main features of autonomous vehicles that need to be represented in models.

Main features to be represented in models	Desired Time-Gap or Desired Gap
	Collision-Avoidance System
	Sensor Detection Range

**Table 2:** $k_{1,\mathrm{setting}}$, $k_{2,\mathrm{setting}}$ and $k_{3,\mathrm{setting}}\;\mathrm{Values}$.

Setting	k_1,setting_	k_2,setting_	k_3,setting_
Very Short (VS)	1.8	8.0	2.52
Short (S)	1.5	6.3	2.07
Middle (M)	1.2	4.7	1.62
Long (L)	0.9	3.0	1.17

**Table 3:** Summary of ten case scenarios.

Case	Non-AV with IDM+	AV with Helly (FACC) Model	Note
No.1	100%	0%	No AV
No.2	95%	5%	*a_max_* = 0.6 m/s^2^ Compositon of Avs’ time gap setting Very short 33% Short 29% Middle 24% Long 14%
No.3	90%	10%	
No.4	85%	15%	
No.5	75%	25%	
No.6	50%	50%	
No.7	0%	100%	
No.8	75%	25%	*a_max_* = 1.4 m/s^2^ Compositon of Avs’ time gap setting is same as No.2 to No.7
No.9	0%	100%	
No.10	0%	100%	*a_max_* = 0.6 m/s^2^ Very short 100%

## Data Availability

Not applicable.
